# Supplementary material for: A Comparison of Feathers and Oral Swab Samples as DNA Sources for Molecular Sexing in Companion Birds
Source: Animals (Basel). 2023 Feb 2;13(3):525. doi: 10.3390/ani13030525 (PMC9913368; doi:10.3390/ani13030525)
Supplement: Supplementary file 1 [file animals-13-00525-s001.zip › animals-2107763-supplementary material s1.pdf]

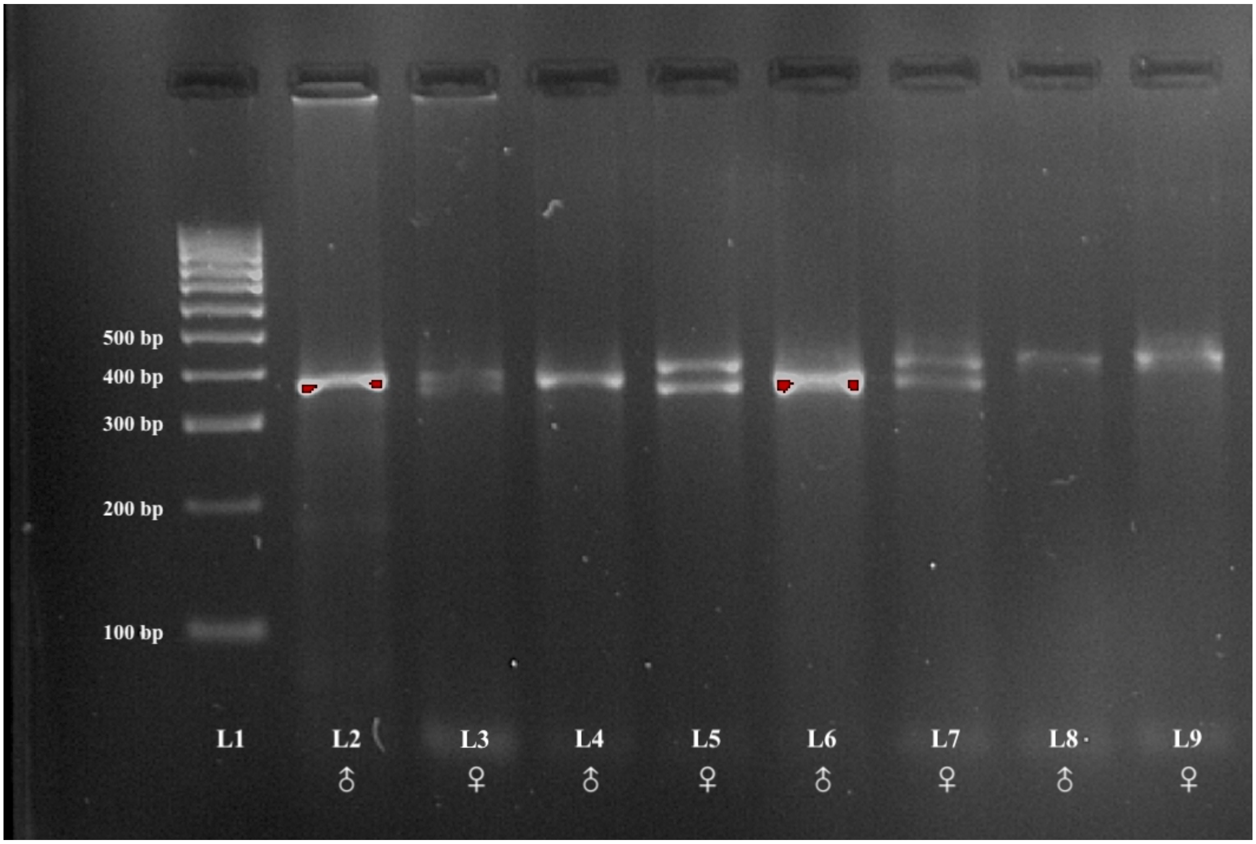

Figure S1. PCR results from oral swabs samples

Legend: L1 – Size standard (100-bp DNA ladder), ♂ - male, ♀ - female, L2 and L3 common pigeons (*Columba livia*), L4 and L5 scarlet-chested parrots (*Neophema splendida*), L6 and L7 rose-ringed parakeets and lovebirds (*Agapornis spp.*).

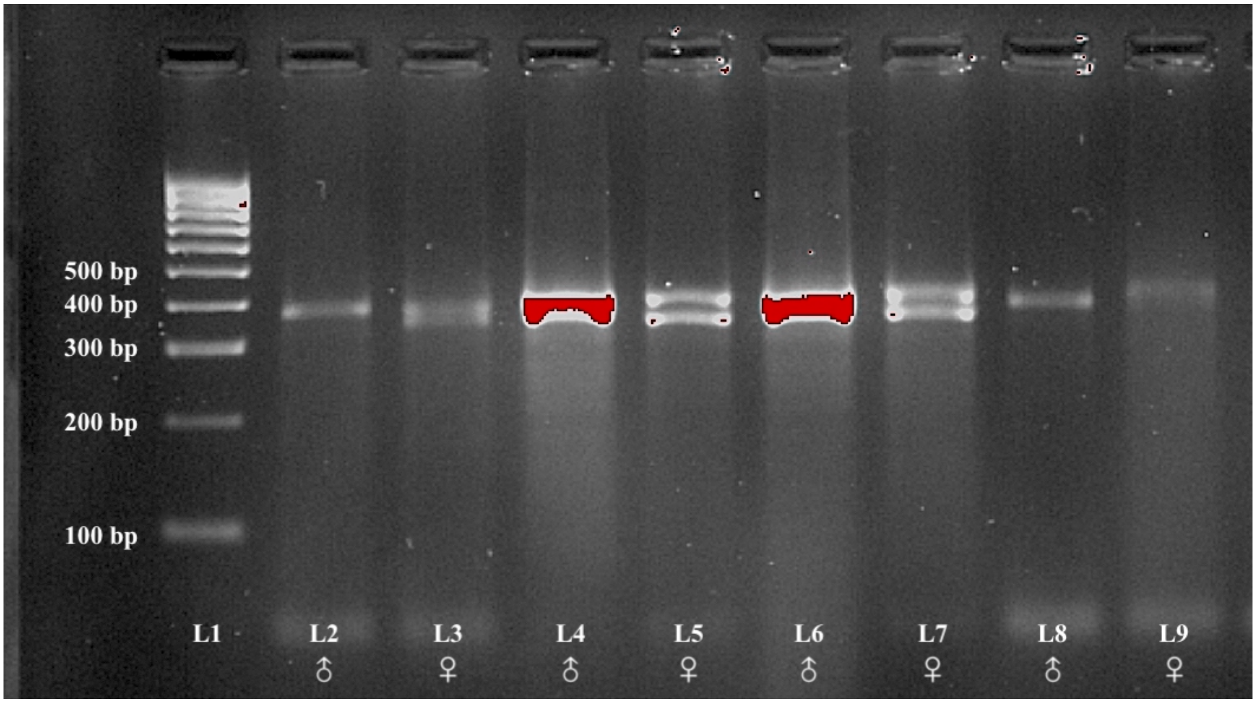

Figure S2. PCR results from feathers samples

Legend: L1 – Size standard (100-bp DNA ladder), ♂ - male, ♀ - female, L2 and L3 common pigeons (*Columba livia*), L4 and L5 scarlet-chested parrots (*Neophema splendida*), L6 and L7 rose-ringed parakeets and lovebirds (*Agapornis spp*.)
